# SEOM clinical guidelines for the treatment of malignant pleural mesothelioma (2020)

**DOI:** 10.1007/s12094-020-02532-2

**Published:** 2021-02-04

**Authors:** E. Nadal, J. Bosch-Barrera, S. Cedrés, J. Coves, R. García-Campelo, M. Guirado, R. López-Castro, A. L. Ortega, D. Vicente, J. de Castro-Carpeño

**Affiliations:** 1 Department of Medical Oncology, Catalan Institute of Oncology, Hospital Duran i Reynals, Avda Gran Via 199-203, l’Hospitalet de Llobregat, Barcelona, Spain; 2 Department of Medical Oncology, Catalan Institute of Oncology, Hospital Josep Trueta, Girona, Spain; 3 Department of Medical Oncology, Vall d’Hebron Institute of Oncology, Hospital Universitari Vall d’Hebron, Barcelona, Spain; 4 Department of Medical Oncology, Hospital Son Llatzer, Palma de Mallorca, Spain; 5 Department of Medical Oncology, Complejo Hospitalario Universitario A Coruña, Coruña, Spain; 6 Department of Medical Oncology, Hospital General Universitario de Elche, Elche, Spain; 7 Department of Medical Oncology, Hospital Clínico Universitario de Valladolid, Valladolid, Spain; 8 Department of Medical Oncology, Complejo Hospitalario de Jaén, Jaén, Spain; 9 Department of Clinical Oncology, Hospital Universitario Virgen de Macarena, Sevilla, Spain; 10 Department of Medical Oncology, Hospital Universitario La Paz, Madrid, Spain

**Keywords:** Malignant pleural mesothelioma, Clinical guidelines, Treatment, Diagnostic

## Abstract

Mesothelioma is a rare and aggressive tumour with dismal prognosis arising in the pleura and associated with asbestos exposure. Its incidence is on the rise worldwide. In selected patients with early-stage MPM, a maximal surgical cytoreduction in combination with additional antitumour treatment may be considered in selected patients assessed by a multidisciplinary tumor board. In patients with unresectable or advanced MPM, chemotherapy with platinum plus pemetrexed is the standard of care. Currently, no standard salvage therapy has been approved yet, but second-line chemotherapy with vinorelbine or gemcitabine is commonly used. Novel therapeutic approaches based on dual immunotherapy or chemotherapy plus immunotherapy demonstrated promising survival benefit and will probably be incorporated in the future.

## Introduction

Mesothelioma is an uncommon but aggressive and highly lethal malignant tumor arising from the serous membranes, being malignant pleural mesothelioma (MPM) the most common entity. Median age of MPM patients is 70 years, with increasing incidence with age and a male:female ratio of 3:1.

The most frequent cause of MPM is asbestos exposure mainly through occupational or para-occupational activities (e.g. cohabitants with exposed industry workers). All the asbestos forms are considered class 1 carcinogenic agents by the International Agency for Research on Cancer (IARC). According to the World Health Organization (WHO), about 125 million people worldwide are exposed to asbestos at their workplaces, and every year more than 107,000 workers die from asbestos-related diseases [1]. In this regard, the WHO recommends establishing registries of people with past and/or current exposures to asbestos and organizing medical surveillance of exposed workers.

Although asbestos were banned by the European Union in 2005, due to the long latency period (usually 20–50 years) among asbestos exposure and MPM diagnosis, the incidence of MPM will peak in Western Europe during the next years. Given this broad interval, banning of manufacture, import or industrial use of asbestos will show benefits in the long term. In addition, environmental exposure is still a major issue and a proportion of deaths might be attributed to asbestos exposure in homes. A replacement plan of asbestos does not exist yet in most European countries. In Spain, asbestos were banned in 2002, but MPM-related deaths will not start to decline in the next decade, despite a discreet lowering in tendency of male mortality from 2001 to 2005, with an expected actual rate of 264 deaths/year [2].

## Methods

Spanish Society of Medical Oncology (SEOM) and Spanish Lung Cancer Group (GECP) defined a panel of medical oncologists to conduct a literature search including systematic reviews, clinical trials, prospective and retrospective observational studies. The Infectious Diseases Society of America grading system was used to assign levels of evidence and grades of recommendation.

## Diagnosis of pleural malignant mesothelioma

### Histopathological assessment in mesothelioma

#### Recommendation

Pathological report should include histological subtype [IA].

##### Literature review

Tissue specimen should confirm the presence of invasion and cytologic samples are not recommended. The 2015 WHO histological classification includes three main subtypes of MPM, epithelioid, sarcomatoid and biphasic, with prognostic importance [3]. A proposal for updating the histologic classification of MPM was conducted by EURACAN/IASLC, which includes more histological subtypes of MPM and further information as architectural patterns, grading and prognostic [4]. Immunohistochemical markers are useful for the diagnosis of MPM and for distinguishing among MPM subtypes from other malignancies but have limited sensitivity and specificity in sarcomatoid subtypes. Epithelioid MPM stains positive to calretinine, cytokeratin 5/6 and WT1 and is negative for CEA, EPCAM, Claudin 4 and TTF1. Loss of BAP1 expression and *CDKN2A/p16* deletion may allow discrimination of MPM from benign pleural lesions. PD-L1 and VISTA were not validated in MPM for its clinical use.

### Genomic testing and next-generation sequencing in mesothelioma

#### Recommendation

Studies based on large-scale genomic analysis have identified molecular subtypes and genetic alterations that may be actionable in the future, but genomic studies are not recommended in routine clinical practice [IIIB]. A proportion of MPM patients carry germline mutations in cancer susceptibility genes, especially those with young age and family history of cancer supporting genetic testing for selected MPM patients [IIIA].

##### Literature review

MPM are dominated by the inactivation of tumor suppressor genes (*BAP1, CDKN2A, NF2, TP53, LATS2* and *SETD2*) and generally have low tumor mutation burden. Although most genomic alterations in MPM are typically considered undruggable, the downstream signaling pathways activated by these mutations, may could be potential therapeutic targets in the future. Germline mutations has been reported in 12% of patients with MPM, but in higher proportion for MPM with early onset or family history [5]. *BRCA1-associated protein (BAP1)* and genes involved in DNA repair are the most recurrent genes.

### Clinical staging in pleural mesothelioma

#### Recommendation

Chest computed tomography (CT) scan with intravenous contrast should be performed as initial evaluation in patients with suspected MPM [IIB]. Tumor staging will be established according to the 8th Edition of AJCC/UICC TNM staging system (Tables 1 and 2). Clinical stage along with other factors (patient fitness, comorbidities, tumor histology) will be crucial to define the level of intervention (curative or palliative intent) in each patient. In patients suitable for surgery, mediastinal biopsy by mediastinoscopy or endobronchial ultrasound (EBUS) are indicated [IIB].

**Table 1:** Definitions of TNM according to the 8th edition of MPM Staging System

Primary tumor (T)	
Tx	Primary tumor not assessable
T0	No evidence of primary tumor
T1	Tumor involving the ipsilateral parietal pleura (including mediastinal and diaphragmatic pleura) with or without involvement of visceral pleura
T2	Tumor involving each of the ipsilateral pleural surfaces (parietal, mediastinal, diaphragmatic and visceral pleura) with at least one of the following features:
	Involvement of diaphragmatic muscle
	Extension of tumor from visceral pleura into the underlying lung parenchyma
T3	Locally advanced but potentially resectable tumor. Tumor involving all of the ipsilateral pleural surfaces (parietal, mediastinal, diaphragmatic and visceral pleura) with at least one of the following features:
	Involvement of the endothoracic fascia
	Extension into the mediastinal fat
	Solitary, completely resectable focus invading soft tissues of the chest wall
	Non-transmural involvement of the pericardium
T4	Tumor involving all of the ipsilateral pleural surfaces with at least one of the following features:
	Diffuse extension or multifocal masses of tumor in the chest wall, with or without associated rib destruction
	Direct transdiaphragmatic extension of tumor to the peritoneum
	Direct extension of tumor to mediastinal organs
	Direct extension of tumor to the contralateral pleura
	Direct extension of tumor into the spine
	Tumor extending through to the internal surface of the pericardium with or without a pericardial effusion, or tumor involving the myocardium

Regional lymph nodes (N)	
Nx	Regional lymph nodes not assessable
N0	No regional lymph node metastases
N1	Metastases in the ipsilateral bronchopulmonary, hilar, or mediastinal lymph nodes (including the internal mammary, peridiaphragmatic, pericardial fat pad, or intercostal lymph nodes)
N2	Metastases in the contralateral bronchopulmonary, hilar, or mediastinal lymph nodes or ipsilateral or contralateral supraclavicular lymph nodes

Distant metastasis (M)	
Mx	Presence of distant metastases not assessable
M0	No evidence of distant metastases
M1	Evidence of distant metastases

**Table 2:** TNM Stage grouping according to the 8th edition of MPM Staging System

Stage	N0	N1	N2
T1	IA	II	IIIB
T2	IB	II	IIIB
T3	IB	IIIA	IIIB
T4	IIIB	IIIB	IIIB
M1	IV	IV	IV

##### Literature review

The clinical manifestations of MPM are not specific and consist of chest pain, dyspnea, fever, excessive sweating and weight loss. Chest radiography might show pleural effusion and/or thickening. Chest CT scan with intravenous contrast is indicated as initial evaluation in patients with suspected MPM. Positron emission tomography (PET)–CT can be helpful to assess pleural lesions and to assess the presence of distant metastases should be considered in patients considered for surgery and prior to talc pleurodesis. Functional magnetic resonance imaging (MRI) may be considered to define T stage. Imaging tests are crucial to guide the optimal biopsy site and to determine clinical staging according to the 8th Edition of AJCC/UICC TNM staging system (Tables 1 and 2). EBUS has higher sensitivity and negative predictive value than mediastinoscopy when assessing nodal invasion in MPM [6].

### Diagnostic method in pleural mesothelioma

#### Recommendation

The diagnosis of MPM should always be based on the results obtained from an adequate biopsy in the context of appropriate clinical, radiologic, and surgical findings [IIA]. In patients with undiagnosed pleural effusion in whom the differential diagnosis includes MPM, a thoracoscopic biopsy should be considered. Alternative and less invasive methods, as image-guided needle biopsies, can be used in unfit patients not suitable for a thoracoscopy.

##### Literature review

Ultrasound-guided thoracocentesis is recommended in patients with pleural effusion. Cytological assessment of pleural fluid has limited sensitivity for MPM diagnosis [7]. Biopsies can be obtained percutaneously under radiological guidance, under direct vision at thoracoscopy (either by local anaesthetic thoracoscopy or video-assisted thoracoscopic surgery [VATS]) or under open pleural biopsy. The biopsy technique will vary depending on the distribution of the disease, the presence of pleural effusion, the suitability for surgery or invasive procedures and their availability. A less invasive alternative for patients with pleural mass, nodular thickening, or unfit for surgery is performing a CT or ultrasound (US) guided core biopsy.

### Methods to assess radiological response in pleural mesothelioma

#### Recommendation

Modified Response Evaluation Criteria in Solid Tumors (mRECIST) criteria version 1.1 is recommended when assessing tumor lesions and tumor response in CT scans and requires the expertise of a radiologist [IIA].

##### Literature review

Response assessment in MPM using conventional RECIST is challenging due to its growth pattern and morphology. In 2004, mRECIST were proposed to assess tumor response in MPM [8] which have been recently updated to version 1.1. Currently, mRECIST are considered the preferred method for tumor measurement, despite they have not been prospectively validated [9].

## Local therapy

### Surgery

The role of surgery in patients with MPM remains controversial, mainly due to limited data available and the lack of standardization to define the best surgical approach, but above all, because surgery has not proven a robust survival benefit in randomized clinical trials. However, surgical resection may be appropriate on carefully evaluated patients in centers with experience in managing MPM.

#### Recommendation

1. For MPM patients with symptomatic pleural effusions candidates to palliative chemotherapy, complete drainage of the pleural space with subsequent pleurodesis is recommended.
  - VATS-PP (partial removal of parietal and/or visceral pleura for diagnostic or palliative purposes) for the management of symptomatic MPM effusions has no effect on overall survival and results in increased complications and longer hospital stay than talc pleurodesis [IA].
  - In patients with lung entrapment, insertion of a tunneled pleural catheter can alleviate respiratory symptoms effectively, although might increase the risk of seeding along the catheter tract.
2. Surgical interventions for maximal cytoreduction with macroscopic complete resection of all tumor (defined as < 1 cm of residual tumor after resection and curative intents) include extrapleural pneumonectomy (EPP) and pleurectomy/decortication (P/D) and should be performed in highly specialized centers by experienced thoracic surgeons. They should only be considered in selected patients with early stage (confined to pleural envelope, no N2 lymph node involvement) and epithelioid histology, given the substantial morbidity and mortality associated with these procedures.
  - EPP involving extrapleural pneumonectomy, *en bloc* resection of parietal pleura, pericardium diaphragm, lung and visceral pleural treatment.
  - EPD with extended pleurectomy/decortication with parietal and visceral pleurectomy, with resection of the diaphragm, and/or pericardium as required may result in lower perioperative mortality than EPP. EPD is the preferred surgical method since has lower respiratory postoperative morbidity and preserves better quality of life. This technique yielded longer overall survival compared with chemotherapy alone in some series.

##### Literature review

Although initial studies suggested that VATS-PP might be superior option to talc pleurodesis, the MesoVATS randomized controlled trial demonstrated no survival advantage with VATS-PP in patients with pleural effusion, which increased length of hospital stay compared with talc pleurodesis [10]. To date, only one prospective, randomized trial, the Mesothelioma and Radical Surgery (MARS) trial, has evaluated the added benefit of performing EPP versus no EPP in the setting of trimodal therapy. No survival benefit was observed, with a median OS of 14.4 months for the EPP group and 19.5 months for the no EPP group, (HR 2.75, *p* = 0.016) and 19% postoperative mortality rate observed in the EPP group [11]. However, these results are controversial due to the small sample size and the fact that OS was not the primary endpoint. Several metanalysis favored EPD over EPP because of the higher mortality after EPP without a survival benefit over EPD [12, 13]. An ongoing randomized clinical trial (MARS 2) should determine whether P/D or EPD after induction chemotherapy leads to superior outcomes compared with chemotherapy alone.

### Radiotherapy

Although mesothelioma cells are moderately sensitive to RT, its utilization is limited by high risk of injury in the lungs and other surrounding organs at risk and because did not improve overall survival.

#### Recommendation

1. Prophylactic RT of chest wall procedure tracts should not be routinely offered [IA].
2. The role of RT as part or multimodality treatment is controversial and due to intensive toxicity should be performed in highly specialized centers by experienced radiation oncologists. The best timing for delivering RT after surgical intervention and/ or in conjunction with chemotherapy should be discussed in a multidisciplinary team.
  - Postoperative RT (45–60 Gy) after chemotherapy and EPP reduce local recurrence but did not improve overall survival. Intensity-modulated radiation (IMRT) therapy seemed particularly promising and can reduce local recurrence after EPP and protect organs at risk. Postoperative RT after P/D is usually not recommended because of toxicity. Hemithoracic IMRT after P/D may be considered in centers with experience.
  - Preoperative RT has not been shown to improve survival and may be considered in a context of clinical trials due to the high risk of severe post-radiation neumonitis. IMRT neoadjuvant therapy followed by EPP may be an option for resectable epithelial MPM.
  - Radical RT to the entire hemithorax used in isolation in the setting of an intact lung and unresectable disease has not been shown to improve survival and is associated with unacceptable toxicity.
  - Palliative RT for localized pain, dysphagia and airway obstruction in MPM can be considered. Although the optimal dose has not been established, for patients with chest pain, doses ranging 20–40 Gy appear to be effective.

##### Literature review

Several randomized clinical trials (RCT) have not shown a reduction in tract metastases with prophylactic RT to chest wall procedure tracts, and did not improve quality of life, chest pain, analgesia requirement and survival [14, 15]. The efficacy of EPP followed by hemithoracic RT has been promising, with in-field failure rates of approximately 15–30%. However, in the SAKK17/04 trial, comparing adjuvant hemithoracic radiation vs no RT in EPP resected patients, no significant differences in terms of overall survival were observed. This trial has several important limitations: early close due to poor accrual and substantial patient dropout from registration to randomization. The use of EPP has gradually declined in recent years in favor of using less radical lung-sparing approaches such as P/D. The hemithoracic intensity-modulated pleural radiation technique (IMPRINT) in conjunction with chemotherapy and P/D demonstrated that a planned does of 50.4 Gy in 23 fractions can be administered safely [16]. Neoadjuvant IMRT followed by EPP has been evaluated in the SMART trial, with a median OS of 51 months and disease-free survival of 47 months in patients with epithelial subtype compared with 10 and 8 months, respectively, for biphasic subtypes [17].

Indications for palliative RT in patients with MPM include pain management, treatment of dysphagia and airway obstruction, and relief of compression of the superior vena cava. RT has also been used to palliate distant metastases, such as those in the bone and the brain. The optimal radiotherapy dose remains unclear; a phase II trial, the SYSTEMS2, is aiming to establish optimal dose/fractionation for symptom control.

## Systemic therapy

### First-line therapy

#### Recommendation

Following multidisciplinary assessment in the thoracic tumors board, patients with MPM who are not suitable for surgery should receive first-line treatment with platinum-based chemotherapy [IA]. In patients who cannot tolerate cisplatin, carboplatin might be used in combination with pemetrexed [IIA]. Maintenance with pemetrexed does not improve overall survival [IIA]. The standard of care consists of 4–6 cycles of cisplatin plus pemetrexed. Chemotherapy should not be postponed and should be considered before clinical deterioration. Clinical trials should be always considered.

##### Literature review

In the EMPHACIS trial, MPM patients not suitable for curative surgery were randomized to cisplatin plus pemetrexed versus cisplatin alone [18]. Combining cisplatin plus pemetrexed improved the response rate (41.3% vs 16.7%), PFS (5.7 vs 3.9 months, *p* = 0.001), overall survival (12.1 vs 9.3 months, HR = 0.77, *p* = 0.02) and patients' quality of life. Similar efficacy results were achieved with carboplatin in non-randomized phase II trials [19]. The MAPS trial evaluated the addition of bevacizumab to standard chemotherapy followed by maintenance with bevacizumab improved overall survival (18.8 vs 16.1 months, HR = 0.77) at the expense of higher toxicity [20]. In the STELLAR trial, a randomized phase II trial, TTFields were delivered to the thorax in addition to conventional chemotherapy in patients with unresectable MPM [21]. Median overall survival was 18.2 months and hematological toxicity constituted the most common grade 3 adverse events. Bevacizumab and TTFields has not been approved by the European Medicines Agency.

### Second-line therapy

#### Recommendation

Participation in clinical trials should be encouraged due to the limited efficacy of second-line chemotherapy. Retreatment with pemetrexed-based chemotherapy can be considered in MPM patients who achieved durable disease control (> 6 months) with first-line pemetrexed-based chemotherapy [IIB]. Second-line pemetrexed is recommended in patients previously treated with first-line chemotherapy regimens that did not include pemetrexed [IA]. Second-line chemotherapy with gemcitabine or vinorelbine can be offered [IIB].

##### Literature review

No therapy has demonstrated to increase overall survival compared to supportive care alone in randomized clinical trials. The limited efficacy of drugs evaluated in second line warrants patients’ referral for participating in clinical trials. Vinorelbine and gemcitabine are used as a second-line treatment in MPM, despite its modest efficacy reported in single arm phase II clinical trials. Distinct immune checkpoint inhibitors have been evaluated in single arm phase II clinical trials with promising efficacy, ie, combination for nivolumab plus ipilimumab (MAPS2) or nivolumab alone (INITIATE, MERIT). However, the PROMISE-meso phase III randomized clinical trial did not demonstrate survival benefit of pembrolizumab over physician-choice chemotherapy (gemcitabine or vinorelbine) [22].

## Future directions

The recent advances in understanding the biology of mesothelioma and the identification of novel potential targets may open new therapeutic avenues.

Preclinical data showed that BAP1 inactivation sensitizes mesothelial cells to inhibition of enhancer of zeste-homolog 2 (EZH2). Tazemetostat, an EZH2 inhibitor, showed promising results in a phase II trial for patients with relapsed MPM.

The most promising results come from clinical trials assessing the combination of immunotherapy with other treatments such as dual immune checkpoint inhibition, Chimeric Antigen Receptor Transduced T cells (CAR-T) or chemotherapy. A phase I trial combining CAR-T cells given intrapleurally and pembrolizumab achieved an overall response rate of 50% in 14 patients with MPM. Further validation of this approach is warranted.

The combination of platinum and pemetrexed with durvalumab in previously untreated patients with MPM has shown promising overall survival (median ranging 18.4–21.1 months) and acceptable safety profile in the DREAM and PrECOG LLC studies [20]. Ongoing phase III clinical trials are evaluating chemotherapy combined with immune checkpoint inhibitors and antiangiogenic therapies. Using a distinct approach, the phase III clinical trial CheckMate-743 compared frontline nivolumab plus ipilimumab with platinum plus pemetrexed in previously untreated MPM, has recently demonstrated significant improvement in overall survival with the dual combination of immune checkpoint inhibitors (18.1 vs 14.1 months, HR = 0.74, *p* = 0.002) [24]. Patients with PD-L1 positive or non-epithelioid histology had larger benefit (HR = 0.69 and 0.46, respectively).
